# Supplementary material for: Upshaw-Schulman syndrome-associated ADAMTS13 variants possess proteolytic activity at the surface of endothelial cells and in simulated circulation
Source: PLoS One. 2020 May 4;15(5):e0232637. doi: 10.1371/journal.pone.0232637 (PMC7197795; doi:10.1371/journal.pone.0232637)
Supplement: S1 Fig — Multimer analysis of pooled plasma (left lane) and recombinant wtVWF (right lane) was performed by SDS–agarose gel electrophoresis and immunoblotting onto a nitrocellulose membrane with luminescent visualization. The figure is composed of one gel. The black line indicates deleted lanes with multimers not relevant for this study. (PDF) [file pone.0232637.s001.pdf]

Supplemental Figure S1

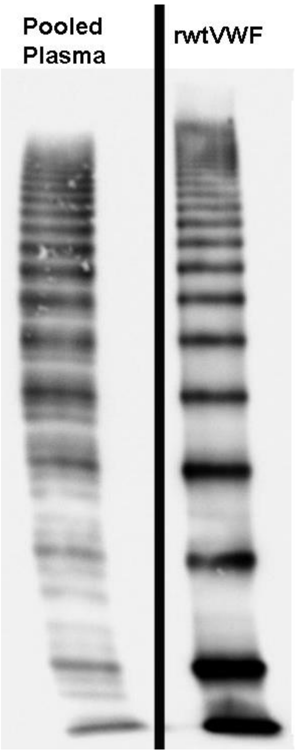

Supplemental Figure S1: Multimer analysis of recombinant wtVWF

Multimer analysis of pooled plasma (left lane) and recombinant wtVWF (right lane) was performed by SDS–agarose gel electrophoresis and immunoblotting onto a nitrocellulose membrane with luminescent visualization. The figure is composed of one gel. The black line indicates deleted lanes with multimers not relevant for this study.
