# Supplementary material for: Upshaw-Schulman syndrome-associated ADAMTS13 variants possess proteolytic activity at the surface of endothelial cells and in simulated circulation
Source: PLoS One. 2020 May 4;15(5):e0232637. doi: 10.1371/journal.pone.0232637 (PMC7197795; doi:10.1371/journal.pone.0232637)
Supplement: S2 Fig — Magnified ROIs of panels (A) and (B) are shown in (C) and (D), respectively. To show the VWF strings, the yellow lines were moved below the strings (above for strings 4, 49 and 63) and the beginning of the strings is marked by red arrows. Strings 13 and 26, which were not cleaved after 10.5 min are marked by a blue arrow (D). (PDF) [file pone.0232637.s002.pdf]

Supplemental Figure S2A. Marked VWF strings before addition of ADAMTS13.

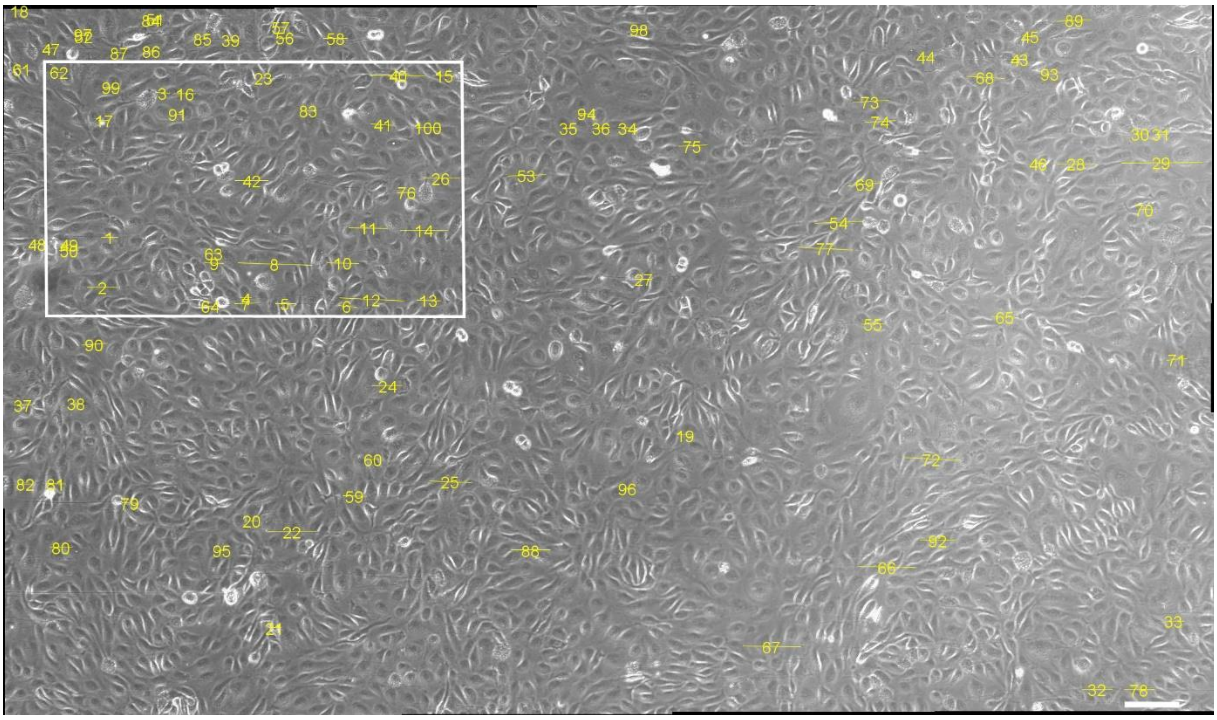

Supplemental Figure S2B. Marked VWF strings 10.5 min after addition of 100 ng/ml wtADAMTS13.

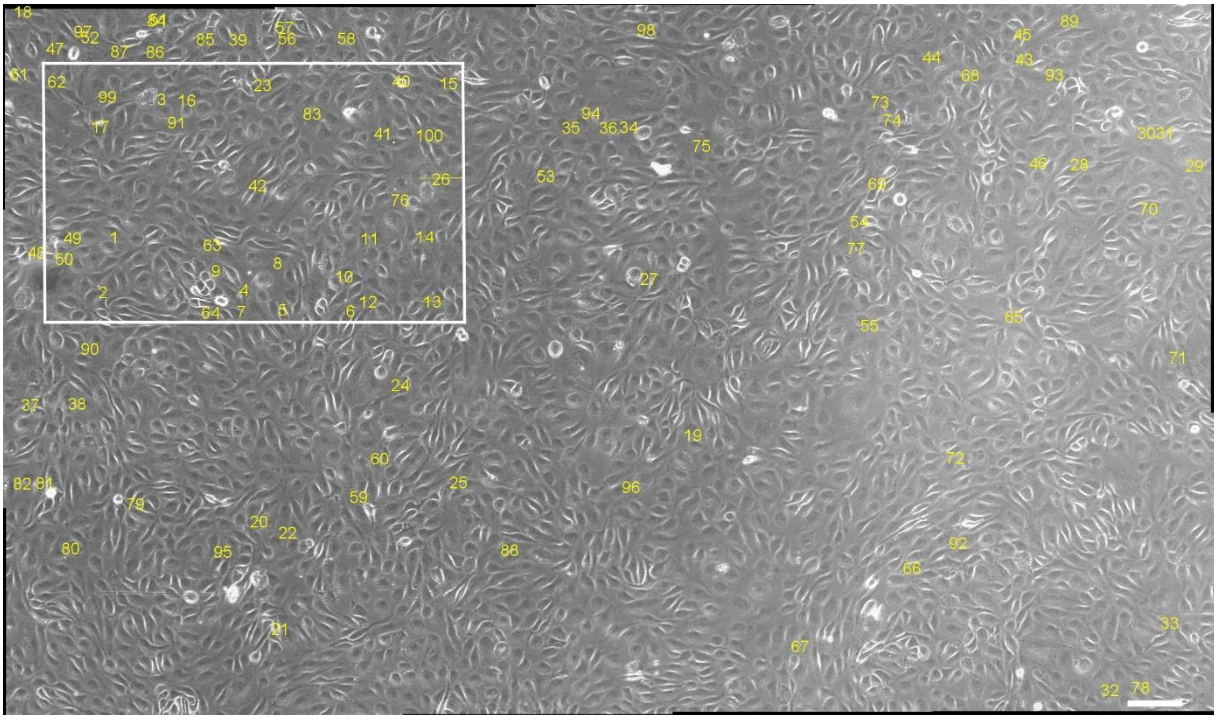

HUVEC were stimulated with histamine and VWF strings were detected using GPIb-latex beads. To determine string length, 100 strings were marked using the ImageJ software (yellow lines) before (A) and 10.5 min after (B) addition of 100 ng/ml wtADAMTS13. To better visualize VWF stings, the ROI marked by a white box is shown magnified in panels (C) and (D).

Supplemental Figure S2C. VWF strings before addition of ADAMTS13.

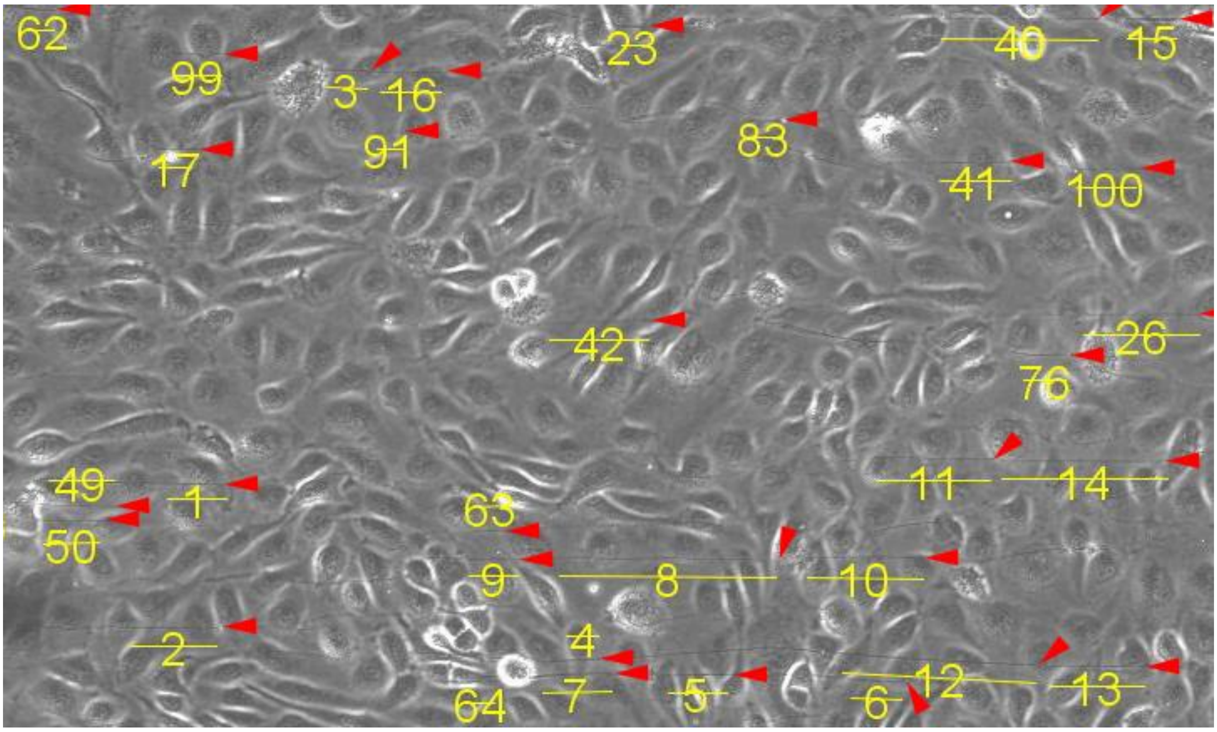

Supplemental Figure S2D. VWF strings 10.5 min after addition of 100 ng/ml wtADAMTS13.

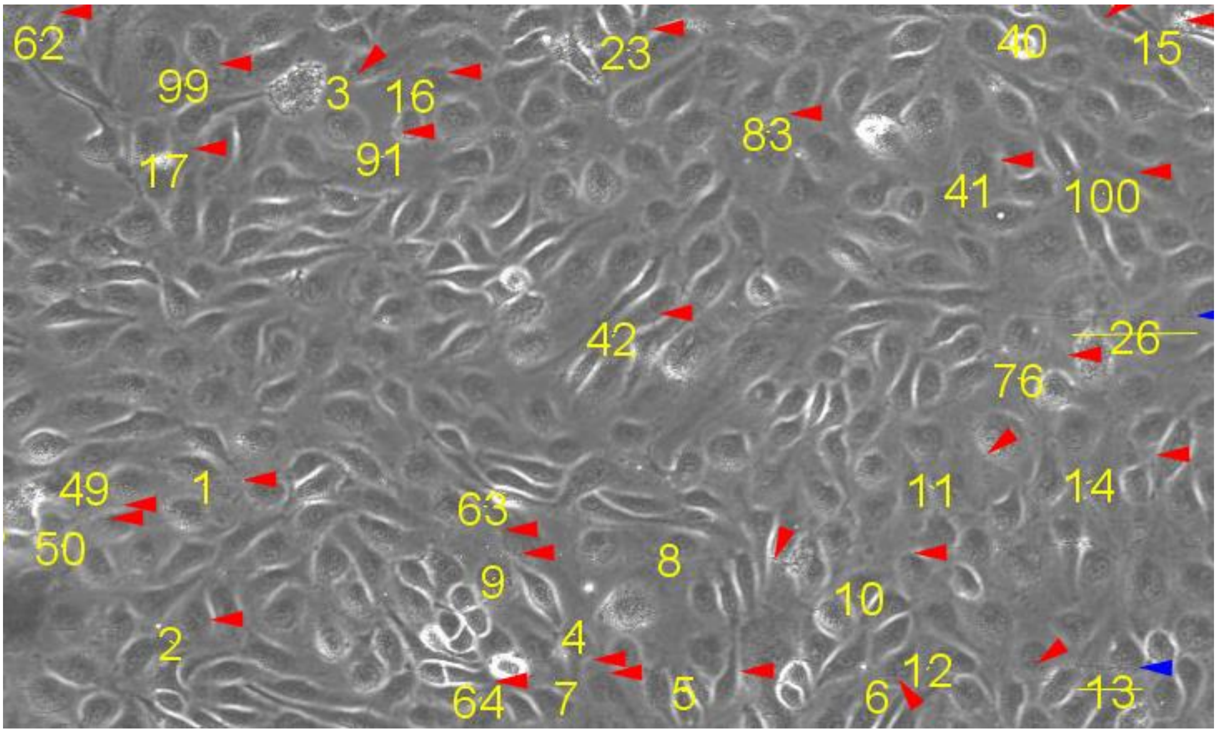

Magnified ROIs of panels (A) and (B) are shown in (C) and (D), respectively. To show the VWF strings, the yellow lines were moved below the strings (above for strings 4, 49 and 63) and the beginning of the strings is marked by red arrows. Strings 13 and 26, which were not cleaved after 10.5 min are marked by a blue arrow (D).
