# Supplementary material for: Upshaw-Schulman syndrome-associated ADAMTS13 variants possess proteolytic activity at the surface of endothelial cells and in simulated circulation
Source: PLoS One. 2020 May 4;15(5):e0232637. doi: 10.1371/journal.pone.0232637 (PMC7197795; doi:10.1371/journal.pone.0232637)
Supplement: S3 Fig — Three representative quantification visualizations of LTA measurements summarized in Fig 4 are shown. The raw signal is depicted in black and the intra-changepoint mean between two consecutive changepoints is represented by horizontal red line segments. Each signal is sampled at two points in time (8 min and 50 min; blue vertical line segments) by calculating the intra-changepoint mean at this point (point of intersection of blue and red line segment). The difference between these two means quantifies the magnitude of degradation. (PDF) [file pone.0232637.s003.pdf]

Supplemental Figure S3.

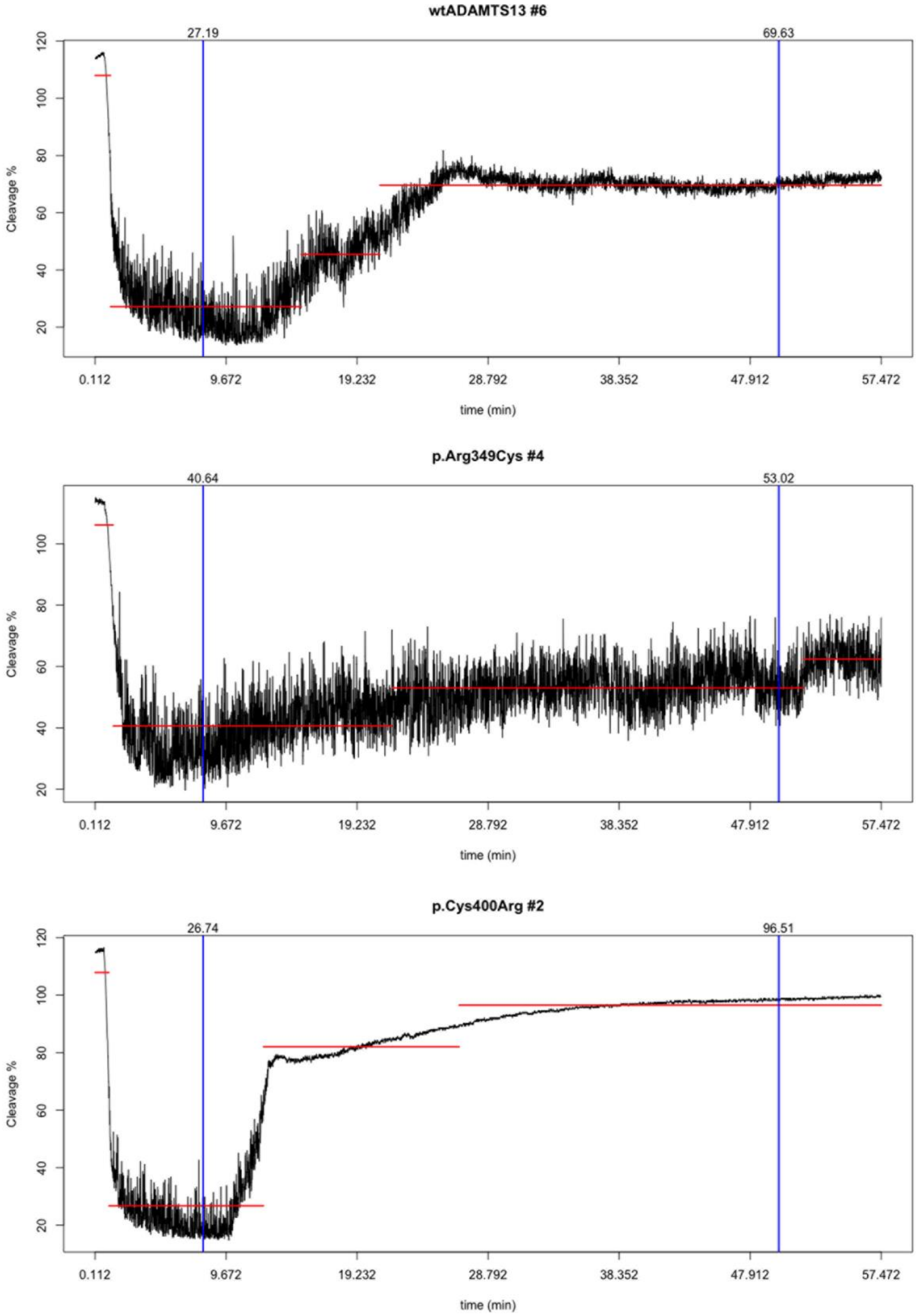

Supplemental Figure S3. Degradation quantification via changepoint analysis using binary segmentation. *Three representative quantification visualizations of LTA measurements summarized in Figure 4 are shown. The raw signal is depicted in black and the intra-*

changepoint mean between two consecutive changepoints is represented by horizontal red line segments. Each signal is sampled at two points in time (8 min and 50 min; blue vertical line segments) by calculating the intra-changepoint mean at this point (point of intersection of blue and red line segment). The difference between these two means quantifies the magnitude of degradation.
